# Attention in natural scenes: contrast affects rapid visual processing and fixations alike

**DOI:** 10.1098/rstb.2013.0067

**Published:** 2013-10-19

**Authors:** Bernard Marius 't Hart, Hannah Claudia Elfriede Fanny Schmidt, Ingo Klein-Harmeyer, Wolfgang Einhäuser

**Affiliations:** 1 Department of Neurophysics, Philipps-University Marburg, Marburg, Germany; 2 Center for Interdisciplinary Research (ZiF), Bielefeld, Germany

**Keywords:** gaze, eye movements, recognition, salience, free-viewing

## Abstract

For natural scenes, attention is frequently quantified either by performance during rapid presentation or by gaze allocation during prolonged viewing. Both paradigms operate on different time scales, and tap into covert and overt attention, respectively. To compare these, we ask some observers to detect targets (animals/vehicles) in rapid sequences, and others to freely view the same target images for 3 s, while their gaze is tracked. In some stimuli, the target's contrast is modified (increased/decreased) and its background modified either in the same or in the opposite way. We find that increasing target contrast relative to the background increases fixations and detection alike, whereas decreasing target contrast and simultaneously increasing background contrast has little effect. Contrast increase for the whole image (target + background) improves detection, decrease worsens detection, whereas fixation probability remains unaffected by whole-image modifications. Object-unrelated local increase or decrease of contrast attracts gaze, but less than actual objects, supporting a precedence of objects over low-level features. Detection and fixation probability are correlated: the more likely a target is detected in one paradigm, the more likely it is fixated in the other. Hence, the link between overt and covert attention, which has been established in simple stimuli, transfers to more naturalistic scenarios.

## 1. Introduction

Two measures are frequently used to probe attention in natural scenes: performance for briefly presented stimuli and gaze allocation. While gaze allocation is referred to as a shift of ‘overt’ attention, performance improvements are taken as evidence for an allocation of ‘covert’ attention to the respective stimulus, independent of the location fixated. The control circuits for gaze-shifts by eye movements and for shifts of covert attention overlap (‘pre-motor theory of attention’ [1]) and in simple detection paradigms shifts in covert attention, indeed, precede eye movements [2]. It is largely unknown, however, whether rapid detection performance and gaze allocation, which are measures that operate on very different time scales, are similarly closely linked when it comes to processing of natural scenes. If so, they should be influenced similarly by variations of stimulus features. Here, we ask whether a feature that is frequently associated with attention, luminance contrast, affects performance during rapid serial visual presentation (RSVP) and gaze during prolonged viewing alike.

### (a) Rapid visual processing

Observers can distinguish images they have seen in a rapid sequence from previously unseen images for presentation durations well under 150 ms [3]. Similarly, the ‘gist’ of a scene is recognized very rapidly [4–6]. It has remained challenging, however, to define gist independently from content perceived ‘in a glance’ [7], which renders statements on its quick perception circular. Hence, rapid visual processing is frequently probed by detecting a certain target category (e.g. animal) in a briefly presented image followed by a mask. Under such conditions, target presence is decodable from electroencephalography (EEG) starting about 120 ms after stimulus onset [4], and from magnetoencephalography (MEG) after 100 ms, at least for face targets [8]. An eye movement to report target presence can occur as early as 120 ms after stimulus onset [5], and even faster for face targets [9].

### (b) Attentional limitations of rapid visual processing

Rapid natural scene recognition is nearly unimpaired by a concurrent attentionally demanding task [10]. Similarly, little impairment is observed if two images have to be processed simultaneously: categorical information for each image is decodable from EEG approximately 150 ms after stimulus onset, irrespective of whether one or two images are processed [11]. Although performance degrades for higher numbers of images [12] and for cluttered images with multiple foreground objects [6], nearly attention-free natural-scene processing is not limited to detection, but extends to more fine-grained tasks, such as gender recognition [13]. Despite this evidence for little attentional demands for the rapid processing of temporally isolated stimuli, several attentional limitations exist when natural scenes are presented in rapid sequence. The attentional blink [14], a processing impairment for a second target if a first target is processed correctly, extends from artificial stimuli to sequences of natural scenes. Rapid recognition, but less so detection, shows an attentional blink [15], which is dependent on target category [16]. Even if only one target is presented in a stream of distractors, then a model for gaze in time-varying stimuli [17] also predicts detection [18]. Together with the link between object categorization and attention models [19], this is a first indication of common mechanisms for gaze and rapid detection.

### (c) Rapid visual processing and image features

Categorical information is contained in an image's amplitude spectrum [20]. The relevance of this global feature for human processing has, however, been challenged [21–23], and local features seem, in general, to be more relevant for natural scene processing [21]. Here, we focus on local features, and because colour contributes little to early stages of detection [24], limit ourselves to luminance contrast.

### (d) Gaze as proxy for attention

Attention is often operationalized by gaze orientation (‘overt attention’). Although the precedence of task over stimulus features is established [25–28], most modelling has focused on stimulus-driven aspects. The ‘saliency map’ [29] implements such a view by stating that regions of high differences within various features (‘contrasts’) will be preferentially attended.

### (e) Low-level features and objects in guiding gaze

While the saliency map predicts gaze well for some stimulus classes [30], and the frequency of its features at the centre of gaze is, indeed, increased compared with control locations [31,32], its mechanistic assumptions have been challenged. First, the tendency to look towards the centre of a stimulus and the bias of putting highly salient items (according to the model) towards the centre of a photograph frequently yield an overestimation of the saliency map's performance [33,34]. Second, decreasing local contrast increases the probability of a region to be fixated [35], rather than increasing it. This result, at first glance, seems to contradict the good prediction of fixations by the saliency map, but becomes understandable if one interprets the saliency-map model as predictor of interesting objects in a scene [36]: in this view, objects rather than features attract gaze [37]. The effect that strong contrast modifications attract attention—irrespective of increase or decrease—can be reconciled with this view: it is a plausible hypothesis that strongly modified regions may become qualitatively different from the background (imagine an extreme case, where the contrast is set to 0 leaving just a grey disc in the case of the greyscale images in Einhäuser & König [35]) and thus may act as object-like items with respect to attracting attention. However, the original data of Einhäuser & König [35] alone are also consistent with a feature-based explanation, such as an increase in texture contrast [38]. How contrast modifications interact with objects already present in the scene for guiding gaze and how they affect (rapid) object detection has remained unaddressed.

### (f) This paper: linking gaze and rapid processing

In three experiments, we assess how the two paradigms—rapid presentation and gaze allocation—relate to stimulus features and each other. In experiment 1, luminance contrast of objects in a scene is modified, and the effect on gaze is measured. Unlike previous studies [35,39], modifications here are applied to objects rather than to random locations. In experiment 2, the same stimuli are used as targets in an RSVP paradigm. In experiment 3, contrast modifications have the shape of an object from one category (e.g. animal), but are superimposed over an image of a different category (e.g. foliage), and gaze is measured. This allows disentangling of the effect of contrast modifications *per se* (shape), from the effects of contrast modifications *of objects* (shape and appearance). The combination of experiment 1 and 3 thereby tests the hypothesis of whether objects or second-order low-level features, such as texture contrast [38], drive attention: if texture contrast or a related low-level measure were solely responsible for the increase of attention to contrast-decreased random locations, then the V-shaped effect of contrast modifications on fixation should prevail for modified objects. If, however, objects are primarily attracting attention, the attracting effect of the contrast decrease should compete with the repulsive effect of a reduced visibility of the object, and thus the effect should weaken for contrast decreases. The combination of experiment 1 and 2 will directly test the hypothesis of whether the two seemingly distinct forms of attention, overt attention in space and covert attention in time, are linked in natural scene processing.

## 2. Methods

### (a) Participants

Twenty-four students of the Philipps-University Marburg participated in the experiments (11 female, 13 male, age: 20–29 years, mean age: 24.0 ± 2.5 years), eight in each experiment. All had normal or corrected to normal vision, were naive to the purpose of the study and had not been exposed to the stimuli prior to the experiment. All gave written informed consent prior to participation.

### (b) Experimental set-up

Experiments were conducted in a light and sound isolated room. Stimuli were presented on a 19.7″ EIZO Flex Scan F77S CRT monitor at 100 Hz and 1152 × 864 pixel resolution with maximum luminance (white) of 33.0 cd m^−2^ and minimum luminance (black) of 0.001 cd m^−2^. Observers were restrained by a chin and forehead rest at a viewing distance of 60 cm. Stimulus size was 30.9° × 23.4° (952 × 714 pixels) in experiments 1 and 3, and 11.3° × 8.5° (341 × 256 pixels) in experiment 2. Stimuli were presented centrally on a grey background, whose luminance was matched to the average stimulus luminance in the respective experiment. Eye position was recorded non-invasively by an EyeLink 1000 (SR Research, Mississauga, Ontario, Canada), using the manufacturer's standard settings for calibration (13 pts), validation, saccade and fixation detection. Presentation and eye-tracker control used Matlab (MathWorks, Natick, MA) and its Eyelink and Psychophysics Toolbox extensions [40–42] (http://psychtoolbox.org/). Data were pre-processed in Python v. 2.7.3 (http://www.python.org) with its numpy, scipy and pylab extension modules. Statistical analysis was performed in R v. 2.14.1 [43] (http://www.R-project.org).

### (c) Stimuli and procedure

#### (i) Experiment 1: overt attention for contrast-modified objects

**Figure 1.**
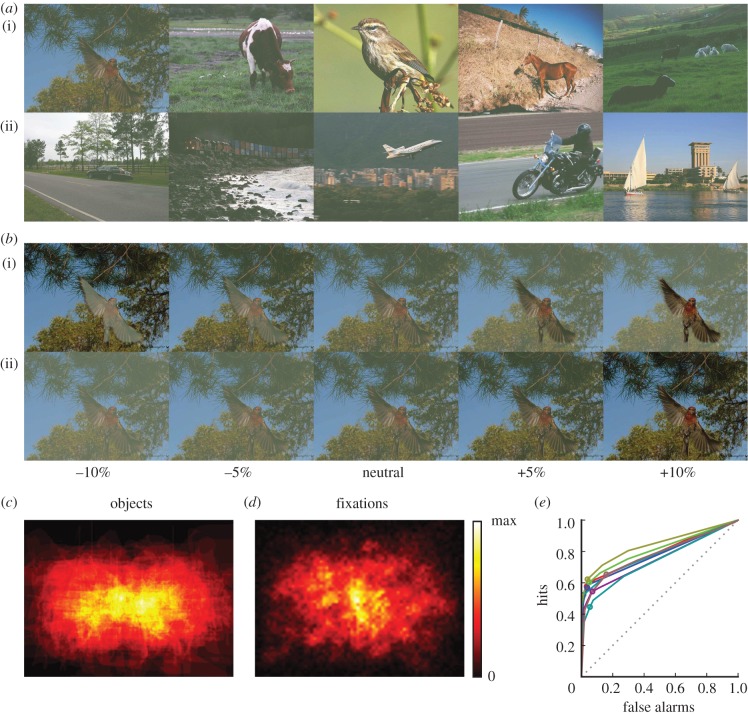
Stimuli and basic behaviour. (*a*) Example neutral stimuli of target category animal (i) or vehicle (ii). (*b*) Stimulus modifications as used in experiments 1 and 2. (i) ‘opposite modifications’. (ii) ‘same modifications’, left 2 columns: negative modifications (−10%, −5%), middle column*:* neutral (identical in both rows, global contrast decrease to 90.9% compared to the original image), right two columns*:* positive modifications (+5%, +10%). (*c*) Two-dimensional histogram of object positions. Displayed size corresponds to stimulus size (952 × 714 pixels, 30.9° × 23.4° visual angle): for each image, pixels are assigned a 1 whenever they are in the target object, or a 0 when they fall in the background; these binary maps are added. Colour map scales from 0 to the maximum entry (35 objects). (*d*) Two-dimensional histogram of fixation locations of experiment 1, aggregated over all neutral stimuli. Histogram is first normalized to unit integral for each stimulus and subsequently averaged over stimuli and observers. Colour map scales from 0 to maximum entry (0.0017). (*e*) Six-point ROC curves for each of the eight observers in experiment 2, based on their confidence ratings (see Methods for details), circle indicates the hit/false alarm rate of each observer based on their answer irrespective of confidence.

Stimuli for experiment 1 were based on 90 images from the PASCAL VOC 2010 database [44], 45 containing one or more means of transportation (‘vehicles’), 45 containing one or more ‘animals’ (birds or mammals; figure 1*a*). Pixels belonging to these ‘target’ objects are defined within the database. The term ‘target’ refers to all objects of the target category in an image, irrespective whether there is one instance (e.g. one animal) or multiple instances (e.g. several animals). The remainder of the image will be referred to as ‘background’.

To modify contrast of objects or images, images were converted to the physiologically defined DKL colour space [45]. All modifications were performed along the luminance axis. Zero on this axis in DKL space corresponds to half the maximum luminance (i.e. 16.4 cd m^−2^). Contrast is modified by scaling around this point, i.e. by subtracting 16.4 cd m^−2^ from the luminance of a pixel, multiplying it with a contrast-change factor (**α**, 2 − **α** or 1/1.1, see below), and then adding 16.4 cd m^−2^ again.

To keep all stimuli within the gamut of the screen, luminance contrast of all images was scaled by a contrast-change factor of 1/1.1 (i.e. 90.9%) prior to any further processing. From each of the resulting 90 *neutral* images (figure 1*b*, middle column), a total of eight contrast-modified versions were created. We modified target contrast and background contrast either in the same or the opposite way (figure 1*b*). When target contrast was decreased, background contrast was either increased (‘opposite modification’) or also increased (‘same modification’); when target contrast was increased, background contrast was either decreased (‘opposite modification’) or also increased (‘same modification’). Technically, in ‘opposite’ modifications, the pixels of the target object were scaled by a contrast-change factor **α** and the pixels of the background by a contrast-change factor 2 − **α**. In ‘same’ modifications, the pixels of the target object and of the background were scaled by the same contrast-change factor **α**. For both modification types, four levels of **α** were used: 0.90 and 0.95 (negative modifications, decrease in contrast), 1.05 and 1.10 (positive modifications, increase in contrast). Throughout the paper, we refer to those as −10%, −5%, +5% and +10%, respectively. With 90 neutral images, four opposite modifications and four same modifications per image, this procedure resulted in 810 stimuli in total.

Observers were presented each of the 810 stimuli once for 3 s. To start a new trial, observers fixated a central fixation spot for 300 ms, after which the image was onset immediately. If observers failed to obtain steady fixation after 5 s, the eye-tracker underwent a recalibration. Observers were instructed to study the image carefully and told explicitly that they were free to look wherever they like during image presentation. The whole experiment was split into nine blocks of 90 trials, organized such that each of the 90 base images was used only once per block. Otherwise, stimuli were ordered randomly. Observers were encouraged to take breaks between the blocks.

#### (ii) Experiment 2: rapid serial visual presentation for contrast-modified objects

The 810 stimuli of experiment 1 served as ‘target’ images for experiment 2. In addition, 553 images from various databases (http://www.photolibrary.uk.com/ and http://visionlab.ece.uiuc.edu/datasets.html) that contained neither target category served as distractors. Distractor images were scaled in luminance contrast by a contrast-change factor of 1/1.1 to span the same dynamic range as neutral target images, but no other modifications were applied.

Observers started a trial by fixating centrally for 300 ms. Immediately afterwards, a 1-s stream of 20 images that either contained 0 or 1 target image was presented at 20 Hz. To avoid primacy or recency effects, the target's serial position was between 6 and 15. Selection and order of distractors were random, but no distractor occurred more than once per sequence. After the presentation, observers were asked to simultaneously report whether the sequence had contained a target and—for computing a six-point receiver operator characteristic (ROC; see below)—the confidence (sure, probable, guess) of their decision by pressing one of six buttons on a standard keyboard. Animal and vehicle targets were tested in separate sessions of 810 trials each (405 target sequences and 405 distractor sequences). Each session was split into 18 blocks, and the same target image did not occur more than once per block. Half of the observers started with the animal session, the other half with the vehicle session.

Behaviour was quantified by signal-detection theory. ‘Hit’ implies correct report of target presence, ‘false alarm’ reporting presence despite actual absence, ‘correct reject’ correctly reporting absence and ‘miss’ reporting absence despite actual presence. To compute a six-point ROC per individual, confidence ratings were used [46]: responses were sorted by confidence (‘present sure’, ‘present probable’, ‘present guess’, ‘absent guess’, ‘absent probable’, ‘absent sure’), and the criterion was shifted along these levels. The ROC's lower left corner corresponds to all responses treated as ‘absent’ judgement (i.e. zero hits, zero false alarms), the first non-trivial point then represents detection performance when only ‘sure present’ responses are counted as present judgement, the second point counts ‘sure present’ and ‘probable present’ as present, and so forth until the upper right point where all ratings are considered as present judgements, resulting in a 100% hit and a 100% false alarm rate. By definition, the middle point of this curve (all absent responses counted as absent, all present responses as present judgement) is identical to the hit/false alarm rate when confidence ratings are ignored (cf. figure 1*e*).

#### (iii) Experiment 3: overt attention, disentangling shape and appearance

Of the 90 images from experiment 1 and 2, 20 animal images and 20 vehicle images were selected at random, in addition to 20 high-resolution natural-scene images from the Tübingen Natural Image Database (http://images.kyb.tuebingen.mpg.de), which contained mostly foliage (figure 4*a*, top). If required, these images were cropped and downscaled using cubic interpolation and were reduced in luminance by a contrast-change factor of 1/1.1.

Twenty triplets of one image per category (animal, vehicle, foliage) were formed. For these triplets, shape and location of the object in one target image (animal or vehicle only) was used to introduce a contrast modification akin to experiment 1 and 2 on the two other images (figure 4*a*), resulting in four stimuli per triplet and modification strength (animal shape on foliage image; animal shape on vehicle image; vehicle shape on foliage; vehicle shape on animal image). Using the four ‘opposite’ contrast-modification levels from experiments 1 and 2 and the neutral stimulus, this procedure results in 20 stimuli per triplet. Note that for the neutral condition, animal shape on foliage and vehicle shape on foliage are identical. In order to have the same number of stimuli for each modification level, however, these duplicates were not removed, such that there were indeed 400 stimuli in total (20 triplets of 20 stimuli). Similarly, a ‘same’ modification would not make the shape visible, but just scale the global contrast of the image, which is unlikely to affect fixation probability on a given object. As this assumption was tested in experiment 1, ‘same’ modifications were not used in experiment 3. The experiment was split into 10 blocks of 40 images each; otherwise, the procedure was identical to experiment 1.

## 3. Results

### (a) Neutral images and basic behaviour

In experiment 1, observers made on average 11.2 ± 1.2 (mean ± s.d. over observers) fixations per neutral image. Although the target covers only 9.5% ± 8.8% of the image's surface area, 56.6% ± 9.3% of fixations fall inside the object (mean ± s.d. over *images*). Targets tend to occur towards the centre of the image (figure 1*c*) as do fixations (figure 1*d*). In addition to such central bias, other confounding factors correlate with the fraction of fixations inside the object (hereafter: ‘fixation probability’); for example, object size (*r*_88_ = 0.49, *p* < 0.001) and the object's root mean-square (RMS) contrast [47] (*r*_88_ = 0.21, *p* = 0.044). This stresses the importance of varying low-level features independently when analysing the effects of contrast on behavioural measures. For neutral images, fixation probability on the object is not correlated to its RMS contrast (*r*_88_ = 0.174, *p* = 0.101). This negative result is probably a consequence of the fact that the RMS contrast of the object in each image (5.18 ± 3.13, mean ± s.d. over images) is indistinguishable from the RMS contrast of its background (5.20 ± 2.58, *t*_89_ = 0.05, *p* = 0.96, paired *t*-test).

In experiment 2, all observers show above chance performance (75.5% ± 2.8% across all stimuli, figure 1*e*). With only 5.7 ± 4.2% false alarms when compared with 43.3% ± 6.0% misses, all observers use a conservative criterion. Unlike fixation probability, hit rate per image (i.e. the fraction of observers who correctly identified the image as target) correlates with the object's RMS contrast across neutral images (*r*_88_ = 0.22; *p* = 0.03). Albeit rather weak, this correlation is a first indication that for detection the contrast of the target image relative to the distractor stream is of importance, a hypothesis that is addressed below.

### (b) Modified images with object modification opposing background modification

**Figure 2.**
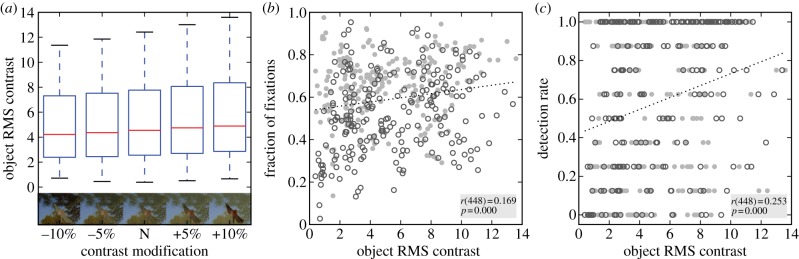
Images with ‘opposite’ modification, effects of luminance contrast. (*a*) Box plots of the object's RMS luminance contrast for the four ‘opposite’ modification levels and the neutral condition (N). In each box plot, the red line depicts the median, the box the inter-quartile range (25–75th percentile) and the whiskers 1.5 times this range (12.5–87.5th percentile). (*b*) Correlation between object contrast and the fraction of fixation falling on it in experiment 1. Data aggregated over neutral images and stimuli with ‘opposite’ modification. Filled circles*:* animals; open circles: vehicles. (*c*) Correlation between object contrast and probability that the target is detected in experiment 2 for the same 450 target images used in (*b*). Discretization of *y*-axis is a consequence of detection rate being number of successful observers in a given image divided by total number of observers, yielding nine discrete levels (0/8, 1/8, … , 8/8). Filled circles: animals; open circles: vehicles.

To verify that contrast modifications are effective, we measure RMS contrast of the object for the five ‘opposite’ modification levels (two positive, two negative, one neutral): despite considerable variability across images that results from the intrinsic variability of contrast across stimuli, the modifications effectively altered luminance contrast (figure 2*a*, *F*_4,89_ = 199.7, *p* < 0.001, repeated measures ANOVA). Across all images with ‘opposite’ or no modification, we find a significant correlation between RMS contrast and fixation probability (*r*_448_ = 0.169, *p* < 0.001, figure 2*b*) and between RMS contrast and detection probability (*r*_448_ = 0.253, *p* < 0.001, figure 2*c*). These correlations persist if vehicle and animal targets are considered separately (fixation, vehicle: *r*_223_ = 0.134, *p* < 0.001; fixation, animal: *r*_223_ = 0.257, *p* = 0.045; detection, vehicle: *r*_223_ = 0.312, *p* < 0.001; detection, animal; *r*_223_ = 0.187, *p* = 0.005). Although the *r*-values—not surprisingly—leave substantial variance in fixation behaviour and detection performance that is not explained by contrast, these data show that for both target categories, detection and fixation probability are significantly influenced by the contrast of the object relative to the background.

### (c) Comparing effects of contrast manipulation on detection and fixation probability

**Figure 3.**
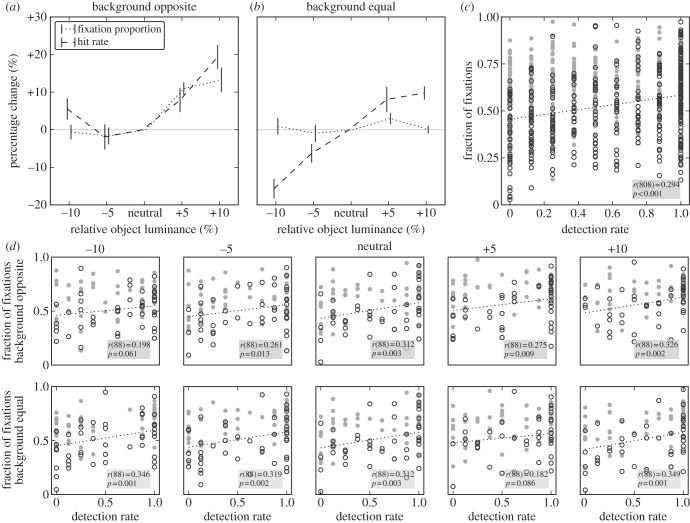
Comparison between experiments 1 and 2. (*a*) Change relative to neutral images for the fraction of fixations on the target (experiment 1, dotted line) and the detection rate (experiment 2, dashed line) for ‘opposite’ modification. Mean and s.e.m. over participants. (*b*) Change relative to neutral images for the fraction of fixations on the target and the detection rate for ‘same’ modification. Notation as in (*a*). (*c*) Correlation over all 810 target stimuli (all modifications) between hit rate in experiment 2 and fraction of fixations on target in experiment 1. Filled circles: animals; open circles: vehicles. (*d*) Data of (*b*) split by modification level, top row*:* ‘opposite’ modification, bottom row*:* ‘same’ modification. Neutral panel included in both rows. Note that (*a*,*b*) normalize data relative to the neutral image for visualization, whereas (*c,d*) use the raw fixation proportions and hit rates, which are also used for all statistical analysis.

To make fixation probability and hit rate commensurate, we normalize both relative to the neutral image and obtain a measure of percentage change relative to neutral. This normalization also removes any variability between images that results from features other than the luminance-contrast modifications, and would therefore affect the neutral and modified versions of an image alike. Such properties, for example, include the size of an object, its centrality with respect to the image (cf. [34]) or general feature-based (dis-) similarity to distractors in experiment 2. When comparing the effects of ‘opposite’ contrast modifications, we find qualitatively similar behaviour between fixation and detection probability (figure 3*a*): if object contrast increases relative to neutral, then fixation and detection probability increase, whereas a decrease has no (fixation) or a slight *positive* (detection) effect. A simultaneous increase of object and background contrast (‘same modification’) has no effect on the object's fixation probability (figure 3*b*, dotted line). This indicates that a general upregulation of contrast in an image does not change the probability that objects are fixated. Average dwell times and the total number of fixations per stimulus are not affected by this same manipulation, either. In contrast to the fixation data, there is a clear effect of simultaneous modifications on detection probability: detection probability decreases if image contrast is decreased, and increases if image contrast is increased (figure 3*b*, dashed line). This indicates that the contrast of the target *image* (i.e. relative to the distractor stream) rather than the contrast of the target *object* is the relevant feature for detection probability in this condition. This effect may also contribute to the slight positive effect for negative contrast modifications on the object (figure 3*a*, left), as for the ‘opposite’ modifications, a negative change to the object implies a positive change to the background. Such a positive change would draw attention to the target *stimulus*, which, in turn, makes detection of the target *object* easier.

### (d) Direct comparison of detection and fixation probability

To directly compare the relation between hit rate and fixation probability for each modification level, we correlate the two measures per image. The two measures are significantly correlated when all data are aggregated (*r*_808_ = 0.294, *p* < 0.001, figure 3*c*), and for animals (*r*_403_ = 0.364, *p* < 0.001) and vehicles (*r*_403_ = 0.390, *p* < 0.001) separately. For 7/9 modification levels, the correlations are significant (figure 3*d*), and this individual significance persists when the alpha-level is adjusted for multiple (nine) tests to an expected false discovery rate [48] of 5%. Taken together, this shows that both behavioural measures are not only driven similarly by object contrast but are also correlated per image, irrespective of the modification applied to object or image.

### (e) Effect of contrast as such (experiment 3)

**Figure 4.**
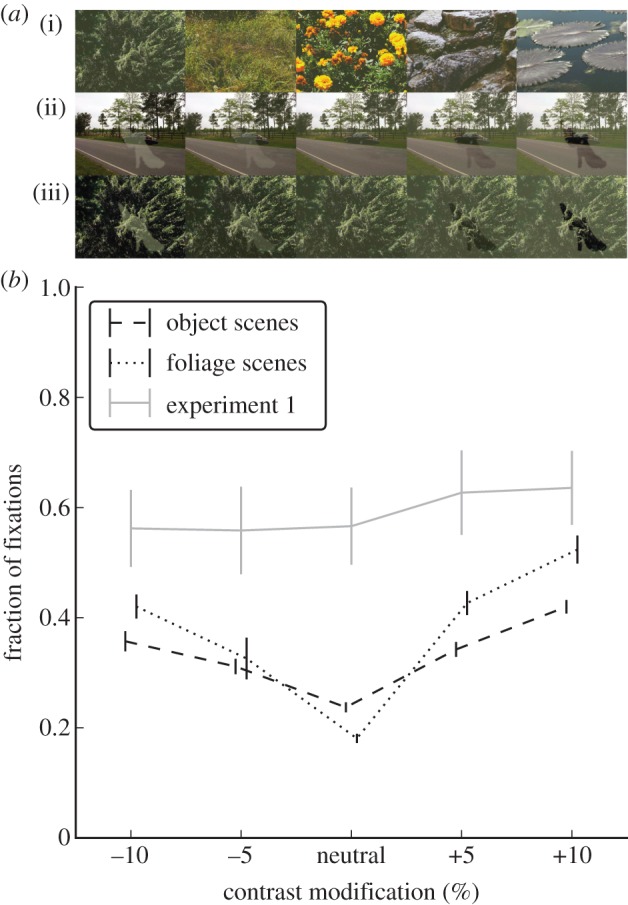
Modifications of contrast detached from modification of object. (*a*) Stimuli for experiment 3: (i) examples of foliage images used in addition to the vehicle and animal database; (ii) animal shape (same as figure 2*a*) superimposed over vehicle image at the five different modification levels; (iii) animal shape superimposed over foliage image at the five different modification levels. (*b*) Dashed line: fraction of fixations on animal/vehicle shape, when superimposed on a vehicle/animal image (mean and s.e.m. over participants); dotted line: fraction of fixations on animal/vehicle shape, when superimposed on foliage image; grey solid line: data from experiment 1 (contrast-modified object) for comparison (same data as figure 3*a* without normalization). Note that the seemingly weak effect in (*b*) when compared with figure 3*a* mostly results from different scaling of the axes; the normalization of figure 3*a* absorbs inter-image variance and enables qualitative comparison to the data of experiment 2; all statistical analysis was, however, performed on the raw data.

Unlike earlier studies [35,39], we here modify an object's luminance contrast rather than a random location. This combines two effects: the effect of contrast as such and a ‘highlighting’ of the object. In other words, the modification of the object's appearance coincides with a change in low-level features within the object's shape. In experiment 3, we test whether the fact that we do not see an attractive effect of negative modifications is a result of these two effects adding up and thus cancelling out for negative modifications. When modifications take the shape of an object from a different scene (figure 4*a*), we indeed observe the V-shaped effect of luminance contrast described earlier (figure 4*b*). There is a main effect of the modification level (*F*_4,28_ = 75.94, *p* < 0.001), a main effect of whether the object shape is applied to an image containing an object or merely foliage (*F*_1,7_ = 10.16, *p* = 0.015), and an interaction between these two factors (*F*_4,28_ = 12.9, *p* < 0.001). This confirms the effects observed earlier for contrast modifications at random locations and furthermore shows that the presence of a true object (appearance and shape) reduces the effect of an object-shaped contrast modification (shape only). In addition, the fact that the V-shaped effect is preserved on foliage images, which contain few or no nameable objects, excludes the possibility that features common to both object categories (e.g. a bias of having the object in the centre and thus the ‘shape-only’ object overlapping with the real object) explain the results. The benefit of tying shape and appearance is further supported by the fact that true objects, whether modified or not, draw consistently more fixations than even the strongest shape-only effect (figure 4*b*). The data of experiment 3 therefore not only reconcile the results of experiment 1 with previous findings but also stress the importance of objects, when compared with their mere low-level defined shape, for attracting attention.

## 4. Discussion

We demonstrate that the probability of an object to be fixated in a scene and the probability of the same object to be detected as target during rapid serial visual processing are correlated across distinct groups of observers. Both measures are qualitatively similarly affected by changes to the object's luminance contrast. These results are in line with the hypothesis that detection during rapid presentation and the allocation of gaze during prolonged viewing tap into the same mechanisms, which are likely to be linked to attention.

### (a) Effect of luminance contrast on overt attention

Although a correlation between fixated locations and contrast during free-viewing [30–32] is largely undisputed, this correlation does not imply a causal drive of attention by contrast [33–35,49]. One key argument against luminance contrast's causal role is the *increase* of fixation probability when contrast of an arbitrary location is sufficiently *decreased* locally [35,39], resulting in a V-shaped dependence of fixation probability on contrast. To reconcile this finding of Einhäuser & König [35] with a low-level feature-based approach to attention, an attractive effect of texture contrast (second-order contrast) on attention was proposed [38]. In this view, texture contrast, which by definition is a V-shaped function of contrast, rather than first-order contrast drives attention. However, the V-shaped effect of contrast has also been in seeming conflict with the positive correlation of fixations with contrast and with the observation that for contrast modifications that ramp gradually over the whole image (either from **α** = −1 at one end to **α** = +1 at the other or between **α** = 0 and **α** = +1 or **α** = −1), fixation probability scales linearly with this contrast modification [50,51]. This linear effect for large-scale ramps was observed notably for the same instruction (‘study the images carefully’) that yielded the V-shaped function of local modifications in Einhäuser & König [35] and is also used here, refuting one concern put forward in Parkhurst & Niebur [38]. Unlike in the previous studies, we here apply contrast modifications to objects. Comparing the data of experiment 1 and 3 thus provides evidence for an alternative possibility to resolve the question as to why fixation probability scales linearly with contrast for global modifications, and is V-shaped for local modifications at random locations: if contrast is bound to an object, a contrast increase also increases fixation probability while a contrast decrease has little effect. If contrast is, however, not bound to an object (experiment 3), then the V-shape re-emerges, even for rather subtle modifications. Taken together, this suggests that local contrast modifications induce an object-like quality, which attracts attention, rather than the contrast modification *per se*. When unrelated to an actual object, these ‘shape-only’ objects compete with actual objects (thus the larger V-shaped effect on foliage), while when tied to an object, an increase in contrast increases fixation probability by increasing the saliency of the object as such. At the present stage, an alternative explanation is still conceivable: negative modifications preserve the attractiveness of the object (even if contrast would be reduced to 0, the object would still be identifiable through its other features, in particular, colour contrasts), whereas the contrast decrease *per se* has little effect (unlike for the local modifications on greyscale images in Einhäuser & König [35] or the shape-only objects). With both interpretations, however, our data provide further support to an object-mediated effect of low-level features [37] and thus for an object-based rather than a feature-based selection process for overt attention [52]. Object precedence over low-level features does not imply that *semantic* object knowledge is required to deploy attention: for example, modified objects (and regions) could also be more easily discriminated from the background and be salient for this reason [53]. In addition, our data show that both shape and appearance of an object are of relevance for attracting attention. In conclusion, experiments 1 and 3 provide further evidence that objects, rather than the low-level features constituting them, are the primary driver of overt attention.

### (b) Effect of luminance contrast on rapid detection

The effect of luminance contrast on detection of targets in rapidly presented sequences is twofold: first, increased contrast of an image (i.e. target and background together) increases detection probability, whereas decreased contrast decreases detection probability. This shows, in line with earlier findings [18], that increased contrast relative to the sequence enhances detection probability. Interestingly, this result is in contrast to results on temporally isolated stimuli, for which contrast modifications of the whole image need to be extreme to show an effect on detection performance [54]. This discrepancy may be interpreted as evidence for the notion that the effects found here arise from attentional limitations in sequential processing rather than from recognition limitations *per se*. Second, increased target contrast and simultaneously decreased background contrast increases detection probability; in turn, for the reverse modification little effect is observed, and if any, the performance improves for lowered target contrasts. The latter is probably a consequence of the negative effect of target contrast and the positive effect of background contrast cancelling each other out in this case. Therefore, the observed effect is consistent with a positive relation between target contrast and recognition performance described earlier for temporally isolated detection [55]. Taken together, performance is improved when contrast is increased, both spatially (target object relative to background) or temporally (target image relative to distractor sequence).

For a given image, its dissimilarity to the set of distractors, for example in terms of low-level features, will affect detection performance, making targets in some images harder to detect than in others [18]. Such differences cannot affect the general pattern described here, as the qualitative effect of contrast modifications holds under normalization to the neutral version of each image (figure 3*a,b*). The difference could add additional variance to the raw performance used for a comparison with fixation data; such would, however, only be able to weaken the correlation between experiments, as experiment 1 is agnostic about the distractors used in experiment 2. Similarly, it cannot be excluded—as in all RSVP studies using this dataset—that the background substantially contributes to target detection as it sets gist and context of the scene. The pattern of results renders a dominant effect of background, however, exceedingly unlikely: compare, for example, performance in the ‘same’ condition at −10% (figure 3*b*) and performance at +10% in the ‘opposite’ condition (figure 3*a*). In these cases, the background, which constitutes the largest part of the image, is exactly the same (figure 1*b*). Nonetheless, detection performance is nearly inverted between these two conditions (+19.3% compared with –15.7% change relative to neutral, figure 3*a,b*). Hence, while background- and target-distractor similarity almost certainly *do* contribute to detection performance, they are uncritical for our present results.

### (c) Comparison of overt and covert attention

Provided a working definition that adopts the notion of attention as selective upregulation of processing resources for some stimuli at the expense of others [56], and the acknowledgement that such selection can occur in either space or time, this paper asks whether both forms of attention share commonalities for natural scene processing. When starting from a purely spatial notion of selective attention, one can rephrase the question equivalently as to whether the observed limitations on rapid processing are ‘attentional’ in the sense that they share properties with spatial attention. For example, when the ‘same’ modification results in experiment 2 are reinterpreted as a more efficient masking of a low-contrast target image embedded in higher-contrast distractors when compared with a high-contrast stimulus in a stream of lower-contrast distractors, the paper shows that such masking has commonalities with spatial attention as expressed by gaze allocation.

For simple stimuli, the selection of saccadic targets (overt attention) and recognition performance among distractors (covert attention) have long been established to share common mechanisms [2]. The pre-motor theory of attention [1] even postulates a common neural substrate for covert and overt attentional shifts. Because outside the realm of natural stimuli RSVP is frequently used as measure of covert attention, and attentional limitations, such as the attentional blink [14], transfer to natural stimulus sequences [15,16], it is likely that our experiment 2 indeed measures a form of covert attention. Under this premise, the present data are the first direct evidence that covert and overt attention exploit similar features also for natural scenes. Even though the observed correlations are not extremely high (of the order of 0.3), they are highly significant for most modification levels. The unexplained variance can, in part, result from sources that by definition influence only one of the experiments (e.g. target–distractor similarity in experiment 2) and from the disjoint sets of observers. Hence, this study is first evidence that the tight coupling between covert and overt attention [2] transfers from simple to complex natural stimuli.

### (d) Implications for real-world processing?

Laboratory eye-tracking experiments, even when using natural stimuli, have limited predictive power for real-world gaze allocation [57]. When observers move actively through real or virtual environments, the functional role of objects (e.g. obstacle versus target [58]), the spatial layout of a scene [59] or even empty locations that have a meaning for action as known from memory [60,61] modulate and supersede saliency as defined by low-level features. In addition, it is at least debatable whether natural perception can or should be decomposed into discrete snapshots [62,63]; and even though saccadic eye movements may provide a natural temporal discretization of perception, the integration over subsequent snapshots remains a challenge [64]. As of now, it remains an open question as to what extent our present data obtained in natural stimuli can be transferred to even more naturalistic—real-world or virtual environment—scenarios. Nonetheless, by demonstrating that two very different measures of attention—covert attention in time and overt attention in space—are correlated in natural scene processing, the present data provide an important step towards bridging the gap between theories and models of attention that are based on comparably simple laboratory stimuli and attention deployment in the real world.

## Notes

All procedures were in accordance with the Declaration of Helsinki and approved by the local ethics committee (Ethikkommission FB04).
